# Supplementary material for: Whole Exome Sequencing Is the Minimal Technological Approach in Probands Born to Consanguineous Couples
Source: Genes (Basel). 2021 Jun 24;12(7):962. doi: 10.3390/genes12070962 (PMC8303193; doi:10.3390/genes12070962)
Supplement: Supplementary file 1 [file genes-12-00962-s001.zip › genes-1252885-supplementary/Supplementary Figures S1-S4.pptx]

## Slide 1
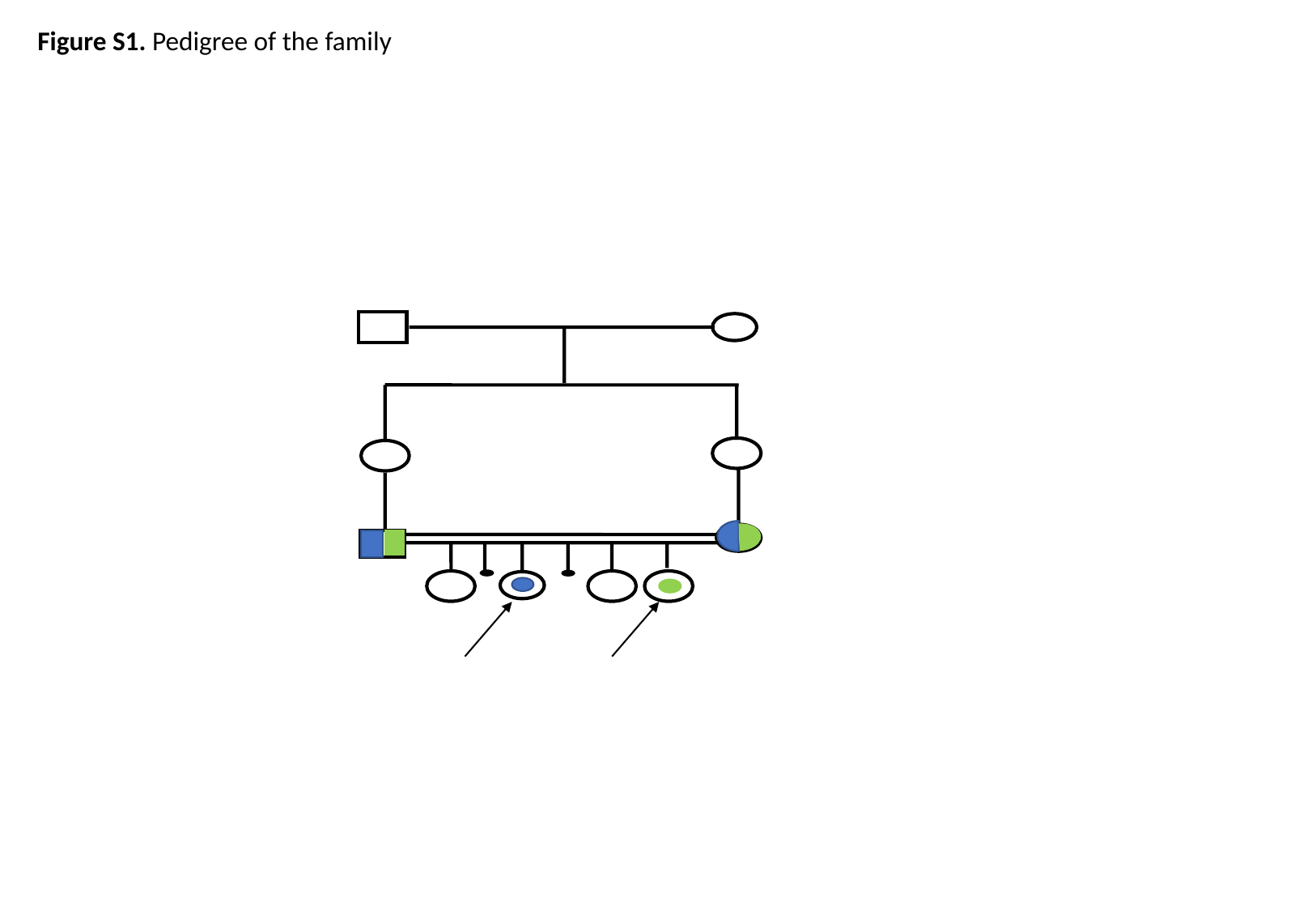

Figure S1. Pedigree of the family

## Slide 2
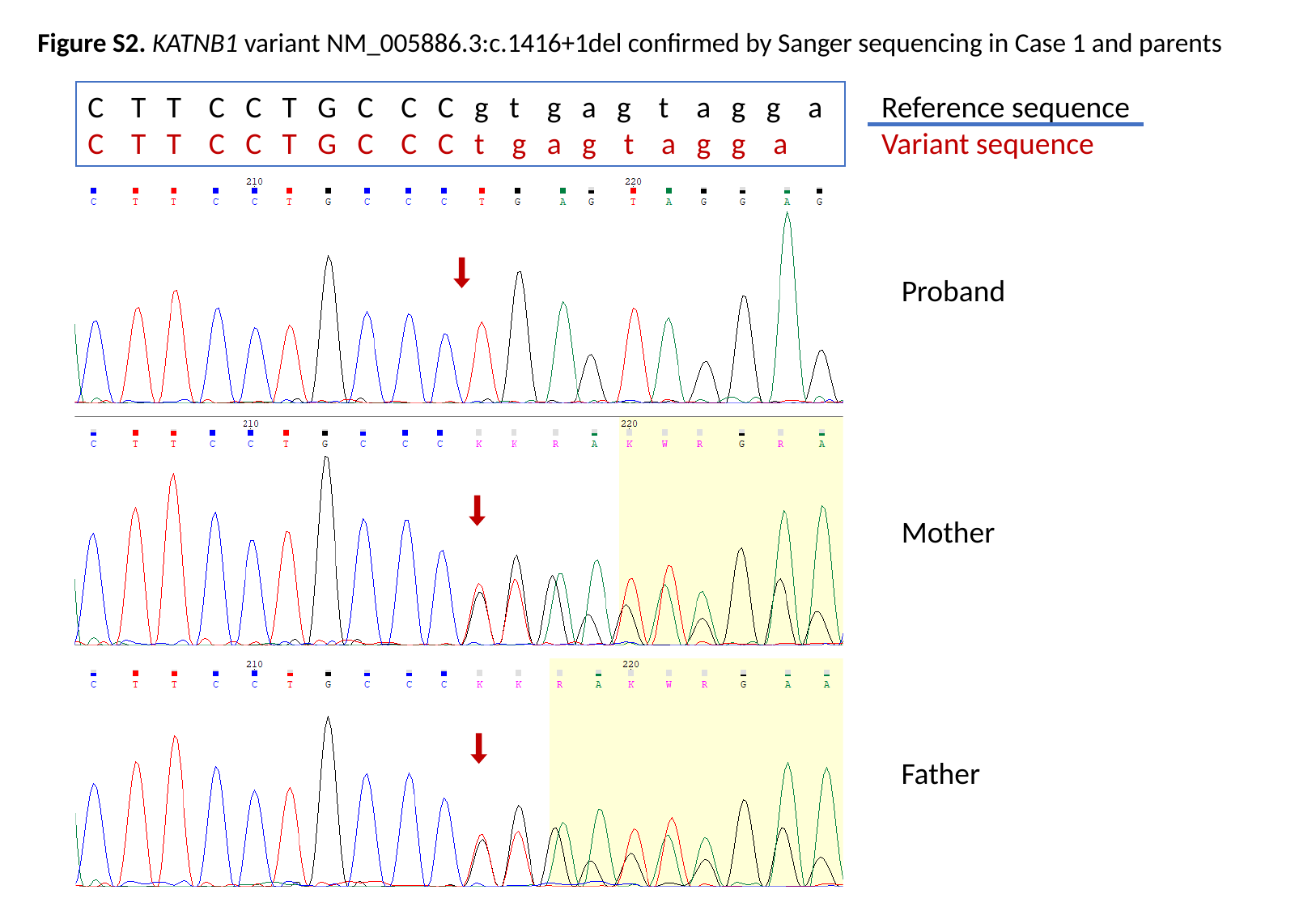

Figure S2. KATNB1 variant NM_005886.3:c.1416+1del confirmed by Sanger sequencing in Case 1 and parents
Reference sequence
Variant sequence
C T T C C T G C C C g t g a g t a g g a
C T T C C T G C C C t g a g t a g g a
Proband
Mother
Father

## Slide 3
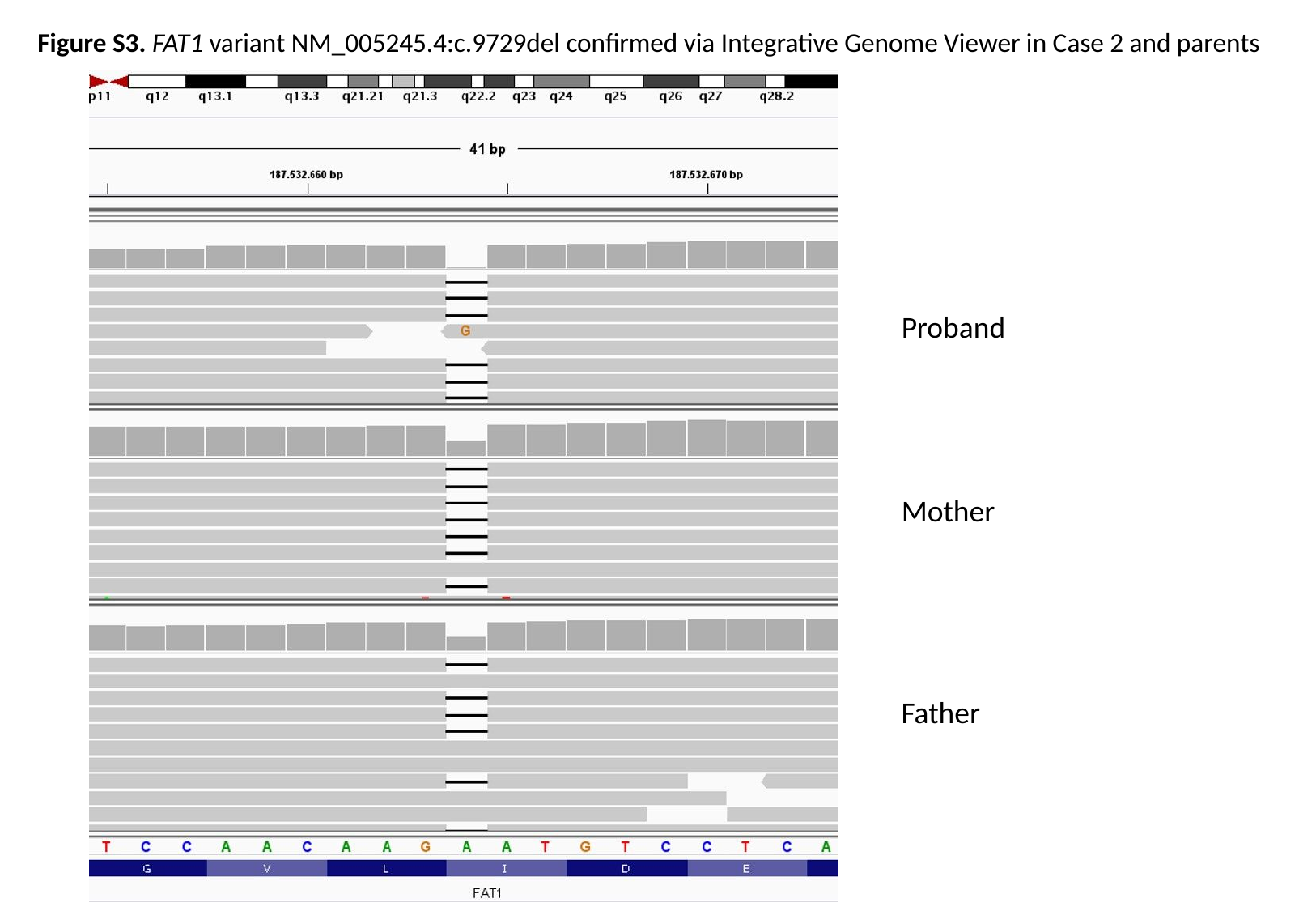

Figure S3. FAT1 variant NM_005245.4:c.9729del confirmed via Integrative Genome Viewer in Case 2 and parents
Proband
Mother
Father

## Slide 4
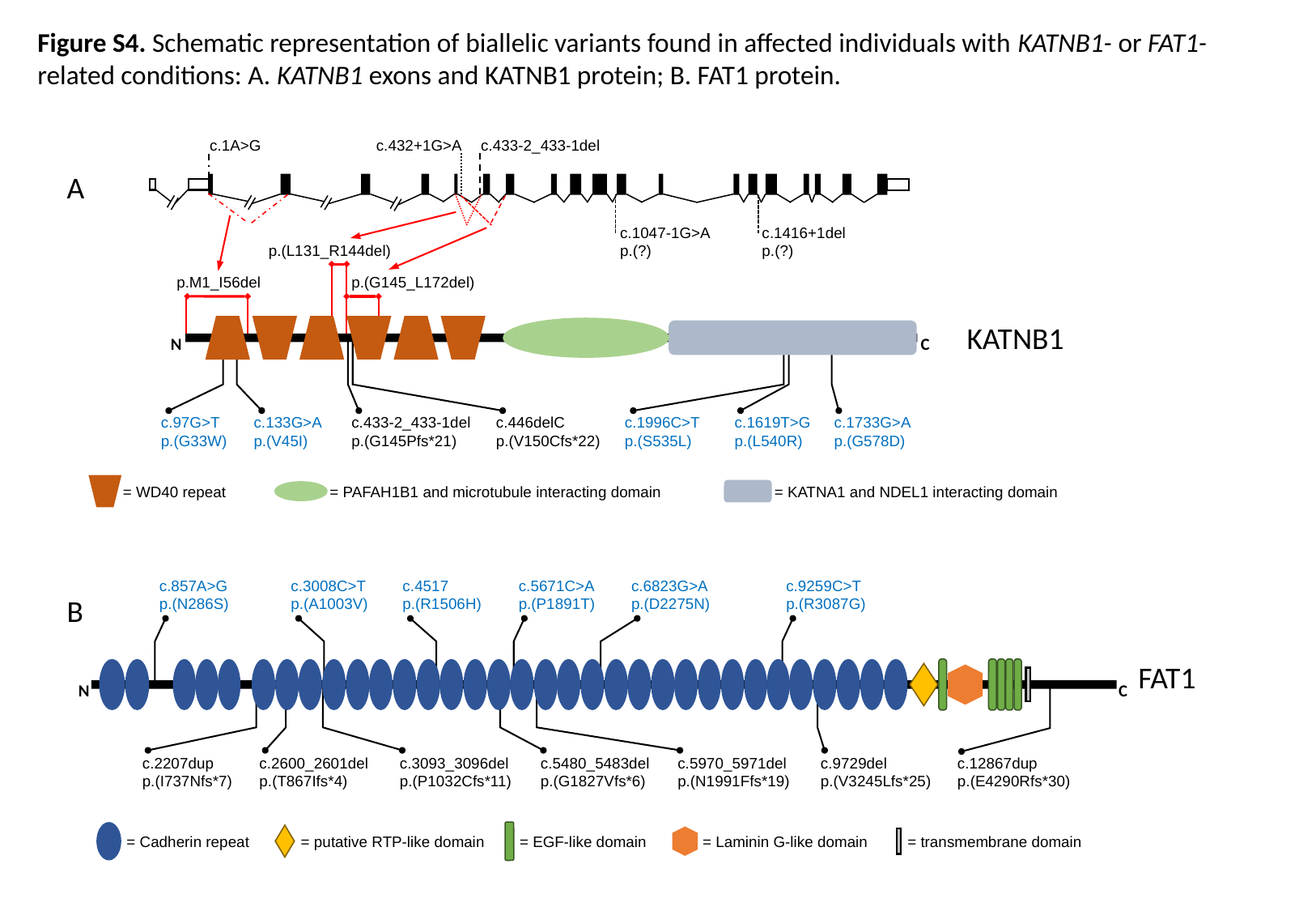

Figure S4. Schematic representation of biallelic variants found in affected individuals with KATNB1- or FAT1- related conditions: A. KATNB1 exons and KATNB1 protein; B. FAT1 protein.
c.1A>G
c.432+1G>A
c.433-2_433-1del
c.1047-1G>A
p.(?)
c.1416+1del
p.(?)
p.(L131_R144del)
p.(G145_L172del)
p.M1_I56del
c.97G>T
p.(G33W)
c.133G>A
p.(V45I)
c.433-2_433-1del
p.(G145Pfs*21)
c.446delC
p.(V150Cfs*22)
c.1996C>T
p.(S535L)
c.1619T>G
p.(L540R)
c.1733G>A
p.(G578D)
= WD40 repeat
= PAFAH1B1 and microtubule interacting domain
= KATNA1 and NDEL1 interacting domain
N
C
A
KATNB1
c.857A>G
p.(N286S)
c.3008C>T
p.(A1003V)
c.4517
p.(R1506H)
c.5671C>A
p.(P1891T)
c.6823G>A
p.(D2275N)
c.9259C>T
p.(R3087G)
c.2207dup
p.(I737Nfs*7)
c.2600_2601del
p.(T867Ifs*4)
c.3093_3096del
p.(P1032Cfs*11)
c.5480_5483del
p.(G1827Vfs*6)
c.5970_5971del
p.(N1991Ffs*19)
c.9729del
p.(V3245Lfs*25)
c.12867dup
p.(E4290Rfs*30)
= Cadherin repeat
= EGF-like domain
= putative RTP-like domain
= transmembrane domain
= Laminin G-like domain
N
C
B
FAT1
