# Supplementary material for: Whole Exome Sequencing Is the Minimal Technological Approach in Probands Born to Consanguineous Couples
Source: Genes (Basel). 2021 Jun 24;12(7):962. doi: 10.3390/genes12070962 (PMC8303193; doi:10.3390/genes12070962)
Supplement: Supplementary file 1 [file genes-12-00962-s001.zip › genes-1252885-supplementary/Table S1_Table S2 - Case 2 WES analysis_VER2.pdf]

| | |
|---|---|
| WES enrichment kit | Twist Human Core Exome Kit (Twist Bioscience) |
| Sequencing platform | NovaSeq 6000 |
| No. reads Patient 2 | 175,404,144 |
| No. reads Mother | 186,141,652 |
| No. reads Father | 179,763,744 |
| Mean Read length | 100 bp |
| Duplication rate | 8.29% |
| Average depth on target | 151X |
| Total number of variants | 105,421 |
| Variants with effect on CDS or affecting splice sites[1] | 9,920 |
| Unknown/low (<0.1%) frequency functional variants | 831 |
| Filtered disease genes (de novo) | 3 |
| Filtered disease genes (recessive trait) | 37 |

**Supplementary Table S1.** WES data output; [1]High quality non synonymous SNPs and indels within coding sequence and splice sites (+/-8).

| Location | Gene | RefSeq | c.DNA | Protein | Max AF | ACM | Effect | Polyphen 2.0 | SIFT | Gene function[1] |
|---|---|---|---|---|---|---|---|---|---|---|
| *De novo* | | | | | | | | | | |
| 1:221057901 | *HLX* | NM_021958.3 | c.1322G>A | p.(Ser441Asn) | - | VUS | Missense | Benign | 0.004 | Th1 lymphocytes maturation (transcription factor) |
| 3:137892399 | *DBR1* | NM_016216.3 | c.267T>G | p.(Asn89Lys) | - | VUS | Missense | Damaging | 0.002 | RNA splicing |
| 20:44869664 | *CDH22* | NM_021248.2 | c.488A>T | p.(Asp163Val) | - | VUS | Missense | Damaging | 0.0 | Brain-specific cadherin (cell adhesion) |
| | | | | | | | | | | |
| *Homozygous* | | | | | | | | | | |
| 1:152058717 | *TCHHL1* | NM_001008536.1 | c.1441G>A | p.(Val481Met) | 0.000147 | VUS | Missense | Benign | 0.128 | transition metal ion binding |
| 5:35871220 | ***IL7R*** | NM_002185.3 | c.442G>C | p.(Val148Leu) | 0.000599 | VUS | Missense | Benign | 0.142 | T cells lymphopoiesis (inteleukin receptor) |
| 5:156566228 | *MED7* | NM_004270.4 | c.215T>A | p.(Phe72Tyr) | 0.000136 | VUS | Missense | Benign | 0.234 | Cofactor for transcription factor activation |
| 9:21187392 | *IFNA4* | NM_021068.2 | c.139A>G | p.(Ile47Val) | 0.000004 | VUS | Missense | Benign | 0.222 | Antiviral immune system activity (interferon) |
| 9:21481259 | *IFNE* | NM_176891.4 | c.435A>T | p.(Lys145Asn) | 0.000799 | VUS | Missense | Damaging | 0.003 | Antiviral immune system activity (interferon) |
| 10:105233226 | *CALHM3* | NM_001129742.1 | c.779G>A | p.(Arg260His) | - | VUS | Missense | Benign | 0.332 | Subunit of ion channel involved in taste perception |
| 11:1579393 | *DUSP8* | NM_004420.2 | c.646A>G | p.(Asn216Asp) | - | VUS | Missense | Probably damaging | 0.032 | Putative regulator of MAPK activity |
| 11:102573534 | *MMP27* | NM_022122.2 | c.569G>A | p.(Gly190Asp) | 0.000004 | VUS | Missense | Damaging | 0.025 | Endometrial-specific matrix metalloproteinase (mestruation) |
| 11:119050726 | *NLRX1* | NM_024618.3 | c.1996G>A | p.(Gly666Ser) | 0.000799 | VUS | Missense | Benign | 0.66 | Antiviral signaling regulation |
| 12:113530981 | *DTX1* | NM_004416.2 | c.956C>T | p.(Pro319Leu) | 0.000004 | VUS | Missense | Probably damaging | 0.008 | Putative regulation of Notch in neurogenesis, lymphogenesis and myogenesis (ubiquitin ligase) |
| 13:41704962 | *KBTBD6* | NM_152903.4 | c.1686C>G | p.(Ile562Met) | 0.000012 | VUS | Missense | Probably damaging | 0.001 | Ubiquitin ligase |
| 13:42142409 | *VWA8* | NM_015058.1 | c.5642T>C | p.(Phe1881Ser) | 0.000998 | VUS | Missense | Damaging | 0.003 | Uncharacterized ATPase |
| 16:12875066 | *CPPED1* | NM_018340.2 | c.265G>A | p.(Gly89Ser) | 0.000241 | VUS | Missense | Damaging | 0.007 | Pro-apoptotic phosphatase |
| 17:7240058 | *ACAP1* | NM_014716.3 | c.5C>T | p.(Thr2Met) | 0.000028 | VUS | Missense | Damaging | 0.037 | Clathrin-dependent export from recycling endosomes |
| 17:7609035 | *EFNB3* | NM_001406.3 | c.119_120insGA GGTGAGTGGCCT | p.(Phe42fs*0) | 0.000044 | VUS | Frameshift | | | Brain-specific ephrin, receptor tyrosine kinases involved in neuronal, vascular and epithelial development |
| 17:80676838 | *FN3KRP* | NM_024619.3 | c.198_199insA | p.(Thr67fs*46) | 0.000521 | VUS | Frameshift | | | Protects proteins from non-enzymatic glycation |
| 22:24951775 | *GUCD1* | NM_001284251.1 | c.19C>T | p.(Arg7Cys) | 0.000014 | VUS | Missense | Benign | 0.0 | Family of proteins involved in GTP conversion to cGMP |
| | | | | | | | | | | |
| *Recessive Compound* | | | | | | | | | | |
| 19:14184539 | *MISP3* | NM_001291291.1 | c.418C>T | p.(Arg140Trp) | - | VUS | Missense | Benign | 0.026 | Uncharacterized protein |
| 19:14184611 | *MISP3* | NM_001291291.1 | c.490C>A | p.(Arg164Ser) | 0.007836 | VUS | Missense | Probably damaging | 0.0 | Uncharacterized protein |

**Supplementary Table S2.** Rare variants of uncertain significance found in case 2. *IL7R* is associated with Severe combined immunodeficiency, T-cell negative, B-cell/natural killer cell-positive type (OMIM #608971), a condition with early manifestations that were absent in case 2. The other genes are not known to be disease-causing. [2]Sources: OMIM, UNIPROT, Human Protein Atlas, GeneOntology
